# Combination of mometasone furoate and oxymetazoline for the treatment of adenoid hypertrophy concomitant with allergic rhinitis: A randomized controlled trial

**DOI:** 10.1038/srep40425

**Published:** 2017-01-18

**Authors:** Wenlong Liu, Lifeng Zhou, Qingxiang Zeng, Renzhong Luo

**Affiliations:** 1 Department of Otolaryngology, Guangzhou Women and Children’s Medical Center, Guangzhou Medical University, Guangzhou, China

## Abstract

In the clinic, approximately 30% of children with adenoid hypertrophy (AH) concomitant with allergic rhinitis (AR) report poor responses to intranasal steroids. To determine whether the combination of mometasone furoate (MF) and oxymetazoline (OXY) is more effective than either agent alone, we performed a two-stage, parallel, randomized, double-blind, double-dummy, clinical trial with 240 AH children with concomitant perennial AR. During the first stage, all children were randomly assigned to the MF or control group for six weeks of treatment. During the second stage, the non-responders from stage one were randomly assigned to 4 groups for 8 weeks of treatment that involved receiving the following treatments: MF/OXY, MF/placebo, placebo/OXY, or placebo/placebo. During the first stage of treatment, 39% of the responders treated with MF achieved greater reductions in total and individual symptom scores than did those on placebo. During the second stage of treatment, the nasal congestion scores of the MF/OXY group significantly decreased. The adenoid/choana ratio of the MF/OXY-treated group decreased and the nasal volume increased significantly. Our results suggest that the combination of OXY and MF is effective and safe for the treatment of AH children with concomitant AR and has a rapid onset of action.

Adenoid hypertrophy (AH) and allergic rhinitis (AR) are the leading factors for nasal respiratory obstruction in children. It has been estimated that 2% to 3% of children may experience apnoea/hypopnea due to obstructive adenoids, and 20–40% of children are affected by AR worldwide^1^,^2^. Moreover, AH is often concomitant with AR, which increases the difficulty and duration of treatment.

Adenoidectomies are commonly considered a definitive treatment for nasopharyngeal obstruction. Nevertheless, this surgical technique has been the subject of some criticism due to its possible negative influence on the systemic immunologic system and postoperative regrowth^3^,^4^. Over the past years, satisfying results have been reported regarding the use of intranasal steroids for nasal chronic obstructive symptoms due to AH in children^5^,^6^,^7^,^8^,^9^. Among the available corticosteroids for clinical use, the safety of mometasone furoate (MF) has been well established in children older than two years^10^,^11^,^12^. Topical MF significantly reduces the size of the adenoid tissue and leads to a supplementary improvement of nasal symptoms. This improvement is achieved in approximately 50–70% of patients, which means that nearly 30% patients still require surgery^5^,^7^,^8^,^13^. We supposed that those who are insensitive to MF or untouchable regarding the target site (adenoid tissue) via MF due to an obstruction caused by a hypertrophic turbinate in the AR mainly contributed to the poor efficacy, especially the latter reason.

Oxymetazoline (OXY) is an adrenomimetic that can agonize the α1 and α2–adrenergic receptors and the endothelial postsynaptic α2 receptors and result in the vasoconstriction of the nasal vascular beds and a volume reduction of the nasal turbinate and thus significantly improve nasal congestion^14^,^15^. OXY has a nearly instantaneous onset of action (5–10 minutes), and the duration of action is between 5 and 6 hours. Whereas the immediate reduction of nasal congestion by OXY is potent, long-term oxymetazoline therapy is hindered by its potential of causing rhinitis medicamentosa^16^. However, if rebound nasal congestion does indeed occur with once-daily dosing, the simultaneous use of an intranasal steroid might be expected to delay its development^17^.

Because of OXY’s rapid onset of action and 5 to 6 hours’ duration of action, we hypothesized that the once-daily at night combination of OXY with MF would provide superior symptomatic relief for AH children with concomitant AR. In this study, we aimed to evaluate the efficacy and safety of the combination of OXY with MF for the treatment of adenoid hypertrophy concomitant with allergic rhinitis.

## Results

### Subjects

Two hundred and forty children were enrolled in this study in the first stage and were assigned randomly to receive MF or placebo. The two groups had comparable ages, sexes, body mass indices (BMIs), baseline symptom scores, adenoidal sizes, nasal volumes, durations of disease, total immunoglobulin E (tIgE) values and eosinophil cationic protein ECP values (Table 1).

During the first stage, 8 patients in the MF group and 11 patients in the placebo group were lost due to different reasons (Table E1). After the first stage, 109 patients in the placebo group and 44 (39%) responders were followed up for six months. During this period, 2 patients in the responders group and 6 patients in the placebo group were lost. In the second stage, 68 non-responders were randomly assigned to four groups with 17 patients in each group. After 8 weeks of treatment and six months of follow up, a total of 5 patients were lost (Table E1).

We compared the baseline demographic data between the responders and the non-responders and between the intra-groups of non-responders, and no significant differences were found except for a lower baseline nasal volume among the non-responders compared with the responders (Table 2).

During the study, the patients received routine medical care for all concurrent illnesses. In the first stage of the study, 9 subjects received antibiotics, including 5 for sinusitis, 2 for bilateral otitis media, and 2 for streptococcal pharyngitis. Four of these subjects received MF, and 5 received placebo. In the second stage, 8 subjects received antibiotics, including 5 for sinusitis, 2 for bilateral otitis media, and 2 for pharyngitis. The frequencies of antibiotic usage among the different groups during the two stages were not significantly different (P = 0.23).

### Compliance and safety

Compliance with the once daily nasal spray was comparable between the treatment periods as assessed by a parental questionnaire and the total weights of the spray bottles that were dispensed during the two stages. The missed does/week (Stage 1: MF *vs* Placebo, 0.5 ± 0.1 *vs* 0.6 ± 0.2 missed does/week, P > 0.05; Stage 2: MF + Placebo *vs* Placebo + Placebo *vs* OXY + Placebo *vs* MF + OXY, 0.6 ± 0.2/0.7 ± 0.2 *vs* 0.7 ± 0.1/0.6 ± 0.1 *vs* 0.7 ± 0.1/0.5 ± 0.2 *vs* 0.6 ± 0.2/0.7 ± 0.1 missed does/week, P > 0.05) and the average consumed MF or placebo (Stage 1: MF *vs* Placebo, 4.0 ± 0.3/3.0 ± 0.2 *vs* 4.1 ± 0.3/3.2 ± 0.4 grams, P > 0.05; Stage 2: MF + Placebo *vs* Placebo + Placebo *vs* OXY + Placebo *vs* MF + OXY, 5.4 ± 0.4/4.3 ± 0.5 *vs* 5.1 ± 0.3/4.2 ± 0.4 *vs* 5.2 ± 0.3/3.8 ± 0.3 *vs* 5.4 ± 0.3/4.1 ± 0.3 grams, P > 0.05) between groups was similar. Additionally, the side effects associated with use of the intranasal aqueous spray were similar for the patients who received the OXY/MF or the placebo (Table E2).

### Symptom scores

**Figure 1:**
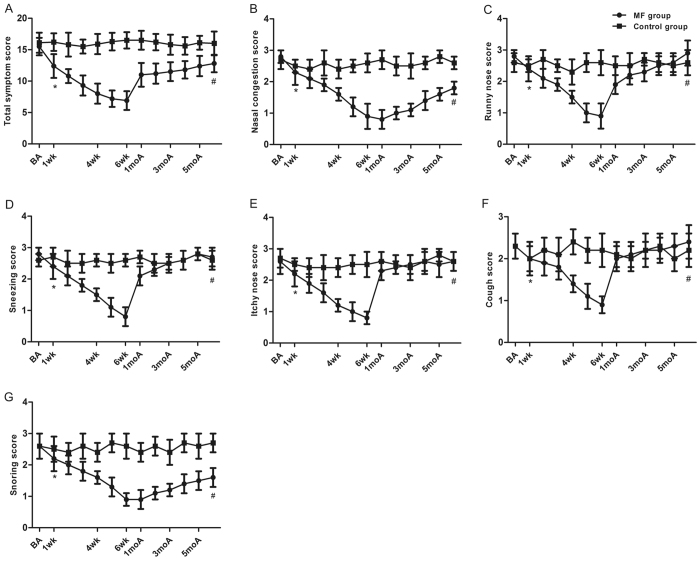
Total and individual symptom scores of different time points between responders and control during the first treatment stage. A for total score and B-G for individual score. *P < 0.05 versus baseline score, ^#^P < 0.05 versus the lowest score during treatment. BA, baseline score; wk, weeks; moA, months after treatment.

After the first stage of the treatment, our results revealed that the treatment with MF led to a greater reduction in the total (6.9 ± 1.5 *vs* 16.5 ± 1.3, *P* < 0.05) and individual symptom scores of the responders than did the placebo (congestion, 0.9 ± 0.4 *vs* 2.6 ± 0.3; snoring, 0.9 ± 0.2 *vs* 2.6 ± 0.4; sneezing, 0.8 ± 0.3 *vs* 2.6 ± 0.2; runny nose, 0.9 ± 0.4 *vs* 2.6 ± 0.4, cough, 0.9 ± 0.2 *vs* 2.2 ± 0.4; itchy, 0.8 ± 0.2 *vs* 2.5 ± 0.4, *P* < 0.05). Moreover, the improvements of the individual and total symptom scores rebounded towards the baseline levels within approximately two weeks after treatment. After six months of follow up, only the congestion and snoring symptom scores were still significant lower than the baseline levels (congestion, 1.2 ± 0.4 *vs* 2.6 ± 0.3; snoring, 1.3 ± 0.2 *vs* 2.6 ± 0.4, *P* < 0.05. Fig. 1). We also found that the runny nose, sneezing, itchy nose and cough scores among the non-responders decreased significantly compared with those of the placebo group (sneezing, 0.8 ± 0.3 *vs* 2.6 ± 0.2; runny nose, 0.9 ± 0.4 *vs* 2.6 ± 0.4, cough, 0.9 ± 0.2 *vs* 2.3 ± 0.4; itchy, 1.0 ± 0.2 *vs* 2.5 ± 0.3, *P* < 0.05) and rebounded to the baseline levels within approximately two weeks after treatment (Fig. E1).

**Figure 2:**
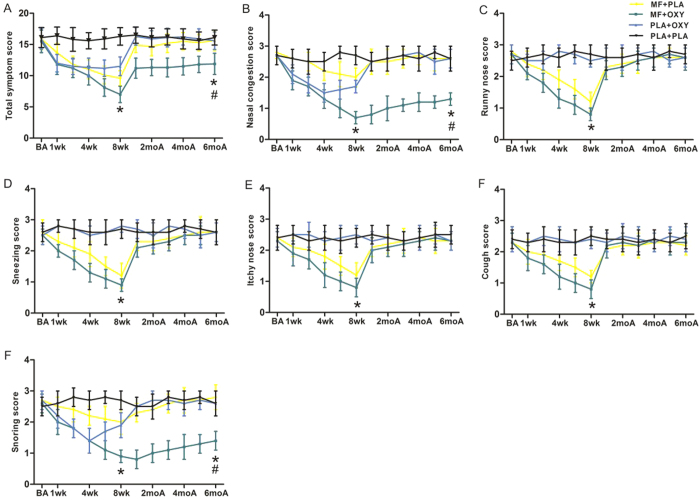
Total and individual symptom scores of different time points among MF + PLA, MF + OXY, PLA + OXY, PLA + PLA group during the second treatment stage. *P < 0.05 versus baseline score, ^#^P < 0.05 versus the lowest score during treatment. A for total score and B-G for individual score. BA, baseline score; wk, weeks; moA, months after treatment.

During the second stage of treatment, our results revealed that both the individual and total symptom scores in the MF/OXY group after treatment were significantly lower than those in the other three groups (P < 0.05). During the six months of follow up, the snoring and nasal congestion scores rebounded slightly but significantly. The scores for the allergy-related symptoms rebounded to the baseline level within approximately two weeks after treatment (P < 0.05; Fig. 2, Table 3). The patients in the MF group exhibited decreased scores for sneezing, runny nose, coughing and itchy nose after the treatment, but they improved and rebounded to the baseline levels within approximately two weeks after treatment (Fig. 2). The scores for nasal congestion and snoring in the MF group exhibited a <50% reduction without obvious subjective improvement (P > 0.05; Fig. 2). The patients in the OXY group exhibited obviously improved nasal congestion and snoring scores one week after treatment, and these improvements lasted for approximately 8 weeks without further improvements. After the cessation of treatment, the nasal congestion and snoring scores rebounded to the baseline levels within two weeks (Fig. 2). Additionally, the sneezing, runny nose, itchy nose and coughing scores in the OXY group exhibited no reductions.

To compare the early effects of the different treatment regimens, we compared the average individual symptoms scores during the first week of therapy in stage two and found that the MF/OXY or OXY treatments resulted in lower nasal congestion and snoring scores than did the placebo or MF treatments (*P* < 0.05), while the allergy-related symptoms exhibited no significant difference between the two groups (Table 4). Our data also revealed that approximately 70% of the patients in the MF/OXY group experienced a nasal congestion score decrease of >50% and a decrease (>50%) in adenoid size within four weeks, whereas approximately 30% of patients required at least six to eight weeks (Fig. E2).

We next compared the individual and total scores for the last week of active treatment to those from the last study week within each treatment arm and demonstrated that there were no significant changes while the patients were on the placebo, and there was a slight increase among the patients on MF/OXY (P < 0.05). Significant increases in nasal congestion and snoring scores were observed in nearly all of the post-cessation treatment days compared with the last week of active treatment with OXY. Significant increases in all of the individual scores and total scores on almost all of the post-cessation treatment days occurred compared with the last week of active treatment with MF. To further test for a lack of a rebound, we compared the first week of active treatment with the last treatment week for all treatment arms and demonstrated that all of the individual scores and total scores were decreased in the MF and MF/OXY groups, whereas only the nasal congestion and snoring scores were decreased in the OXY group (Table 3).

### Adenoid size and nasal volume

**Figure 3:**
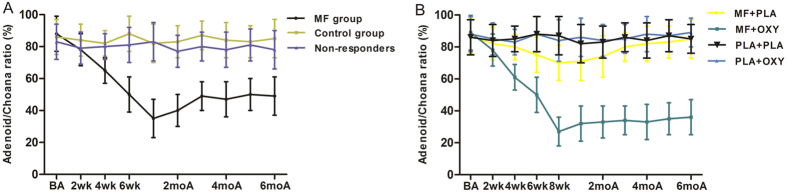
Adenoid/choana ratio of different time points between responders and control during the first treatment stage (**A**) and the ratio of different time points among MF + PLA, MF + OXY, PLA + OXY, PLA + PLA group during the second treatment stage. *P < 0.05 versus baseline adenoid/choana ratio. BA, baseline score; wk, weeks; moA, months after treatment.

**Figure 4:**
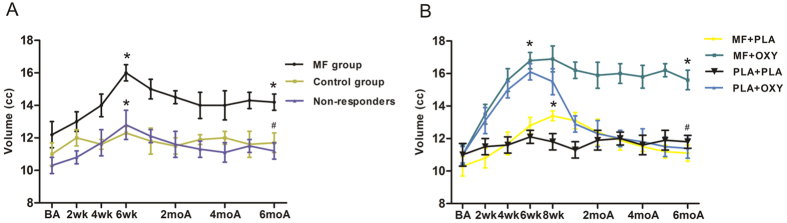
Nasal volume of different time points between responders and control during the first treatment stage (**A**) and the nasal volume of different time points among MF + PLA, MF + OXY, PLA + OXY, PLA + PLA group during the second treatment stage. *P < 0.05 versus baseline nasal volume, ^#^P < 0.05 versus the highest nasal volume during treatment. BA, baseline score; wk, weeks; moA, months after treatment.

After the first stage of treatment, the adenoid/choana (A/C) ratio of the responders in the MF group dropped from 88.2 to 35.1% with a decrease of 53.1% (P < 0.05), and no change was found in the placebo or non-responder group (Fig. 3). Furthermore, the nasal volume of the responders in the MF group increased from 12.2 ± 0.8 cm^3^ to 16.0 ± 0.6 cm^3^ (P < 0.05), and no change was found in the placebo group. Moreover, the improvement of the nasal volume and the A/C ratio of the responders lasted for at least for six months without a significant rebound. In the MF group, the nasal volume increased from 10.3 ± 0.5 cm^3^ to 12.8 ± 0.5 cm^3^ (P < 0.05; Fig. 4).

After the second stage of treatment, the A/C ratio of the MF/OXY-treated group dropped from 87.2 to 61% with a decrease of 26.2% after 4 weeks of treatment. Between the 4th and 8th weeks, the A/C ratio further declined to 27.3%. Six months after treatment, the A/C ratio was 28% without a rebound (P < 0.05; Fig. 3). Regarding the other groups, the changes in the A/C ratio at different time points were not significantly different.

Consistently, the nasal volume of the MF/OXY-treated group increased from 11.2 ± 0.7 cm^3^ to 15.6 ± 0.9 cm^3^ after 4 weeks of treatment. Between the 4th and 8th weeks, the nasal volume further increased to 16.8 ± 0.8 cm^3^. Six months after treatment, the nasal volume was 15.2 ± 0.7 cm^3^ and without a significant rebound (*P* > 0.05). The patients in the MF group exhibited an increased nasal volume after the treatment (10.6 ± 0.6 cm^3^ compared with 13.4 ± 0.3 cm^3^, P < 0.05), and the rebound was significant after the follow-up (13.4 ± 0.3 cm^3^ to 11.1 ± 0.4 cm^3^, P < 0.05). The patients in the OXY group exhibited an obviously improved nasal volume one week after treatment, and this improvement lasted approximately 8 weeks without further improvement (11.0 ± 0.5 cm^3^ to 15.5 ± 0.6 cm^3^, P < 0.05). After the cessation of treatment, the nasal volume rebounded to the baseline level within two weeks (15.5 ± 0.6 cm^3^ to 11.4 ± 0.6 cm^3^, P < 0.05). Regarding the control group, the changes in nasal volume at the different time points were not significantly different (Fig. 4).

## Discussion

AH is the main reason for nasal obstruction and snoring. In the past two decades, the use of topical nasal steroids has brought significant improvement for AH patients, and thus many children have avoided adenoidectomy^18^,^19^,^20^,^21^,^22^. In 1995, Demain and Goetz described the first successful use of beclomethasone for AH in paediatric patients with a 29% decrease in the A/C ratio on average^5^. In 2006, Cengel and Akyol assessed the efficacy of MF for the treatment of AH and found a significant decrease in the adenoid mass of 67.2% in the study group^7^. Another study emphasized that 4 months of treatment with an MF aqueous nasal spray results in successful results at 28 months of follow up^13^. In general, approximately 50–70% of patients achieve 20–50% decreases in adenoid size and significant improvements in symptoms. Additionally, the improvements may last as long as 2 years. However, there are at least two limitations to the previous studies. First, most of the previous studies have only described the characteristics and treatment efficacy of the responders, while they have neglected the analysis of the non-responders. Second, AH children with concomitant AR have not been studied, therefore the effects of AR on the use of nasal steroids is not clear.

In our clinical experience, many AH children with poor responses to MH have concomitant AR. Target sites that are untouchable (i.e., adenoid tissues) by MF due to obstructions caused by hypertrophic turbinates in the AR provide the main contribution to poor efficacy^23^. Therefore, we used the combination of MF and OXY for these children and obtained good efficacy according to our data.

MF was chosen for its low systemic availability, its lack of effects on growth, and its lack of effect on the hypothalamic-pituitary-adrenal axis in children. OXY has been demonstrated to have various antioxidant and anti-inflammatory properties in addition to its potent decongestive effect^24^,^25^. Previous studies have demonstrated that the concomitant administration of MF and OXY may provide better improvements in AR patients, especially those with nasal congestion symptom^26^,^27^,^28^,^29^,^30^,^31^. Therefore, the initial thought of this study was that this decongestant would improve airflow and nasal volume and that MF may act on the turbinate and AH tissue more fully and quickly.

During the first stage of our study, approximately 40% of the AH children with concomitant AR exhibited good responses to MF, and the improvements in nasal congestion and snoring lasted for at least six months with a slight rebound. Among the non-responders, only allergy-related symptoms (i.e., runny nose, sneezing, itchy nose and cough) were controlled during treatment despite the scores returning to the baseline level after treatment. These results are consistent with those of previous studies, which suggests good roles of MF in the treatment of AR despite the short maintenance period. We also found a significant lower nasal volume in the non-responders than the responders, which suggests that the mechanical obstruction of the nasal cavity was the main factor that led to the poor responses of MF in the AH and AR treatments.

To reduce nasal obstruction, we used a combination of MF and OXY. In the MF/OXY group, all of the symptoms and the objective performance were significantly improved, and the onset time was quicker than that in the MF group.

In the MF group, the allergic symptoms were significantly relieved to a similar extent as that observed in the MF/OXY group. Interestingly, the nasal congestion and snoring scores also significantly improved after 8 weeks of treatment, but the reduction in the AH size was far less than 50% (<20%). We also found that a longer time of use of MF led to a longer maintenance period of efficacy and a slower rebound. These results suggest that MF could be solely used in the treatment of AH children with concomitant AR, but it may take effect over a longer time, and the effect might not be obvious. Therefore, further studies are needed to evaluate the long-term use of MF in the treatment of AH children with concomitant AR.

In the OXY group, the nasal congestion and snoring symptoms were relieved within one week. To further test for a lack of rebound, we compared the first week of active treatment with the last week after the cessation and demonstrated that the nasal congestion and snoring scores were not significantly different. These results indicate that the intermittent usage of OXY is safe for AH patients.

Additionally, the recovery from the rebound nasal congestion associated with rhinitis medicamentosa after the cessation of topical decongestants can be hastened by the use of intranasal steroids^32^. Thus, the absence of rebound congestion in our study was expected because of the once daily dosing with OXY, and in the combination group, the MF may have additionally acted to prevent rhinitis medicamentosa.

Additionally, our data demonstrated that the nasal volume was improved during the treatment and followed by improvements in AH size and symptoms. We suppose that the improvement of the nasal volume by MF/OXY caused quick nasal flow and an enlarged nasal space and thus more MF was spread on the AH tissue and acted effectively, which reduced the AH size and ultimately improved the symptoms.

There are also some limitations in our study. First, the sample size was small, and the follow-up period is not sufficiently long. Second, the children who were enrolled in our study were those with severe nasal congestion. Therefore, the efficacy of MF and OXY in the treatment of AH children with mild AR was not explored. Third, the efficacies of MF and OXY in the treatment of AH children with seasonal or non-allergic rhinitis were also not discussed. In the future, clinical trials with large sample sizes and long-term follow-ups are needed to prove the effect of MF/OXY in the treatment of AH children with different types of rhinitis.

In summary, our results suggest that the combination of MF and OXY may significantly and safely improve the subjective and objective symptoms of AH children with concomitant AR. The efficacy lasted for at least six months without a significant rebound or rhinitis medicamentosa.

## Methods

### Study population

Between April and September 2014, 240 children affected by both AH and AR were recruited. Informed consent was obtained from either the parents or legal guardians of all study participants, and the study was approved by the institutional review board of Guangzhou Women and Children’s Medical Center, including any relevant details in the methods section. Additionally, all of the experiments were performed in accordance with the relevant guidelines and regulations. The study is registered at http://clinicaltrials.gov (#NCT 02559440, registered on 09/21/2015). At enrollment, all children met the following inclusion criteria: (1) adenoid occluding at least 75% of the nasopharynx at nasal endoscopy, (2) age between 6 and 12 years, (3) chronic obstructive nasal symptoms consistent with AH and AR lasting no less than 12 months, (4) no previous adenoid surgery, (5) moderate-to-severe perennial AR diagnosed by positive skin prick and significant symptoms with a total nasal symptom score of at least 8 of 12 and a congestion score of 2 or 3 during screening. Children with seasonal AR, concomitant tonsillar hypertrophy, upper respiratory infection within the last 2 weeks, sinonasal anatomic anomalies or diseases, craniofacial malformations such as labiopalatal clefts, genetic diseases (i.e., Down’s syndrome), neurologic or cardiovascular diseases, immunodeficiency, history of epistaxis, asthma, hypersensitivity to MF or OXY, or undergoing intranasal, topical, or systemic steroid or antibiotic treatment within the past 4 weeks were excluded.

A skin prick test with a standard aeroallergen panel that included dust mites, animal dander, cockroaches, and mould was performed. TIgE and ECP were also tested using an electrochemiluminescence method with an ELX-800 system and a Unicap system, respectively.

### Study design

We performed a two-stage, parallel, randomized, double-blind, double-dummy, clinical trial with 240 AH children with concomitant perennial AR. During the first treatment stage, 240 children were randomly assigned to the MF (50 μg, 1 puff in each nostril every evening) or control group (normal saline) after a two-week run-in period. After 6 weeks of treatment, the children in the MF group were evaluated and grouped as responders or non-responders according to their improvement of subjective symptoms and objective performance. The responders were defined as the patients whose nasal congestion score decreased >50% and exhibited a decrease (>50%) in adenoid size after treatment such that adenoidectomy could be avoided. The non-responders were those patients whose nasal congestion score decreased <50% and had subjective symptoms, especially nasal congestion, that were not obviously relived.

The responders were followed up for six months without treatment and reassessed. The non-responders underwent a 2-week washout period and were randomly assigned to 4 groups that received the following treatments: MF (50 μg, 1 puff in each nostril every evening)/placebo, MF/OXY (0.05%, 1 puff in each nostril every evening), placebo/placebo, or placebo/OXY. All of the participants received one week “on” OXY or placebo and one week “off” (every other week) for eight weeks. Subsequently, the patients were followed up for six months without treatment, and the evaluations were performed at different time points.

The nasal sprays were labelled with patient code numbers, and the investigator assigned the patients in a sequential randomized fashion to a study code number in blocks of four. The individual nasal spray bottles were identity-masked such that both the patients and the researchers were blind to the treatment assignments. The study blinding was preserved at the study sites until all subjects had completed the study, and the database had been locked. A minimal sample size of 10 patients per arm in this parallel design study was estimated based on an α error of 0.05, a β error of 0.2, an initial mean nasal congestion score of 2.7, and a potential decrease in the nasal congestion score of 50%, while allowing for a 20% attrition rate. Compliance with the medications was assessed both by a parent questionnaire and by a measurement of the drug weight administered. The bottles were weighed before dispensing and returned after treatment. The total drug administered by the patient was calculated as the difference in the bottle weights.

### Evaluation of the subjective symptoms and objective performance

The severities of sneezing, runny nose, nasal congestion, and itchy nose among the AR patients were recorded in the morning and the evening on a 0 to 3 scale. For nasal symptoms, scores of 0, 1, 2, or 3 were defined as none (no symptoms present), mild (mild symptoms that did not interfere with any activity), moderate (slightly bothersome symptoms that slightly interfered with activity/night time sleep), and severe (bothersome symptoms that interfered with activity/night time sleep), respectively. Additionally, cough and snoring were evaluated using a similar scoring system ranging from 0 to 3.

One examiner, in a double-blinded evaluation, calculated the adenoid area in relation to the nasopharyngeal area with a nasopharyngoscope under sedation. For this purpose, videos were analysed using Sony Vegas software, and the images of the adenoid tissue and nasopharyngeal areas were quantitated in pixels with ImageJ software.

Acoustic rhinometry, a quantitative measurement of nasal volume, was performed with an ECCOVISION acoustic rhinometer (Hood Laboratories Pembroke, Mass). Three measurements were made on each side and averaged.

### Time points of follow-up

At stage one, the patients returned to the clinic every week for a total of 8 weeks (two times for the run-in period) for review of their symptom diaries, adenoid size and performance on acoustic rhinometry. At stage two, the responders returned to the clinic six times for evaluations over six months for follow-up (once per month). The non-responders were reassessed at weeks 1, 2, 4, 6, and 8 during 8 weeks of treatment, and they were evaluated six times over a six months follow-up period (one per month).

### Statistical analysis

The primary outcome was the symptom score of nasal congestion. Other outcomes were symptoms of runny nose, sneezing, itchy nose, snoring, cough, total symptom score, nasal volume and adenoid size.

Because the scores were not normally distributed, we compared the scores among treatments by Kruskal-Wallis ANOVA followed by Mann-Whitney testing for post hoc analysis. The nasal volume was normally distributed and analyzed by use of ANOVA with Bonferroni testing for post hoc analysis. To examine the data for a possible rebound effect after cessation of therapy, we performed a Friedman ANOVA on the average symptom score of last week’s treatment and the last study week’s treatment. Further, to make certain that there were no significant rebound effects, we compared average symptom score of the first week of treatment to the last week’s treatment by using the Wilcoxon signed-rank test.

## Additional Information

**How to cite this article**: Liu, W. *et al*. Combination of mometasone furoate and oxymetazoline for the treatment of adenoid hypertrophy concomitant with allergic rhinitis: A randomized controlled trial. *Sci. Rep.* **7**, 40425; doi: 10.1038/srep40425 (2017).

**Publisher's note:** Springer Nature remains neutral with regard to jurisdictional claims in published maps and institutional affiliations.

## Supplementary Material

Supplementary Figures

## Figures and Tables

**Table 1:** Demographic characteristic of children between MF and placebo group.

Group	MF group	Control
Number	112	109
Sex (Male:Female)	53:59	50:59
Age (months)	94.2 ± 28.0	98.3 ± 26.8
Total score	15.5 ± 1.4	16.1 ± 1.3
Nasal congestion score	2.8 ± 0.2	2.7 ± 0.3
Itchy nose score	2.6 ± 0.2	2.5 ± 0.3
Sneezing score	2.7 ± 0.3	2.6 ± 0.4
Rhinorrhea score	2.6 ± 0.3	2.5 ± 0.3
Snoring score	2.7 ± 0.2	2.6 ± 0.2
Cough score	2.2 ± 0.1	2.4 ± 0.1
Adenoid/choana ratio (%)	89.2 ± 10.1	87.2 ± 11.2
Nasal volume (cm^3^)	12.5 ± 0.7	12.2 ± 0.8
BMI (kg/m^2^)	18.7 ± 2.3	21.9 ± 3.0
ECP (ng/ml)	29.2 (4.0–104.0)	28.9 (3.6–119.0)
TIgE (IU/ml)	383.2 (120.5–1002.0)	496.0 (167.3–1170.0)
Duration of disease (years)	1.6 ± 0.7	1.5 ± 0.8

Numbers were presented as mean ± standard deviation or mean (range).

**Table 2:** Demographic characteristic of responders and non-responders and intra-groups of non-responders finished the study.

	Stage 1			Stage 2		
	Responders	Non-responders	MF + Placebo	MF + OXY	Placebo + OXY	Placebo + Placebo
Number	44	68	15	16	16	16
Sex (Male:Female)	21:23	33:35:00	8:07	7:09	8:08	9:07
Age (months)	91.4 ± 22.0	93.1 ± 26.8	93.8 ± 22.5	98.3 ± 23.9	97.2 ± 21.0	95.2 ± 24.5
Total score	16.1 ± 1.4	15.9 ± 1.3	15.9 ± 1.8	16.5 ± 1.5	16.3 ± 1.6	15.3 ± 1.5
Nasal congestion score	2.7 ± 0.3	2.8 ± 0.2	2.6 ± 0.3	2.7 ± 0.3	2.5 ± 0.3	2.6 ± 0.4
Itchy nose score	2.5 ± 0.2	2.4 ± 0.3	2.6 ± 0.2	2.5 ± 0.4	2.5 ± 0.3	2.4 ± 0.3
Sneezing score	2.7 ± 0.3	2.5 ± 0.4	2.6 ± 0.3	2.5 ± 0.3	2.6 ± 0.3	2.4 ± 0.3
Rhinorrhea score	2.4 ± 0.3	2.5 ± 0.3	2.5 ± 0.3	2.6 ± 0.3	2.4 ± 0.3	2.5 ± 0.2
Snoring score	2.6 ± 0.4	2.7 ± 0.2	2.7 ± 0.2	2.6 ± 0.2	2.7 ± 0.3	2.6 ± 0.2
Cough score	2.4 ± 0.3	2.3 ± 0.2	2.2 ± 0.3	2.3 ± 0.4	2.2 ± 0.3	2.4 ± 0.2
Adenoid/choana ratio (%)	85.4 ± 11.0	87.2 ± 11.3	86.1 ± 12.0	83.4 ± 11.1	86.4 ± 11.5	85.1 ± 11.2
Nasal volume (cm^3^)	12.2 ± 0.8	11.0 ± 0.6*	10.5 ± 0.6	11.2 ± 0.4	11.3 ± 0.5	11.1 ± 0.4
BMI (kg/m^2^)	19.2 ± 2.3	20.8 ± 3.0	18.3 ± 3.3	22.4 ± 2.0	21.6 ± 2.3	20.9 ± 3.1
ECP (ng/ml)	39.2 (4.0–104.0)	29.9 (3.6–119.0)	32.2 (3.6–119.0)	41.9 (3.6–119.0)	36.8 (3.6–119.0)	31.2 (3.6–119.0)
TIgE (IU/ml)	393.2 (120.5–902.0)	459.0 (167.3–870.0)	383.2 (188.3–1010.0)	406.0 (213.3–854.0)	515.2 (237.3–970.0)	386.0 (245.3–970.0)
Duration of disease (year)	1.7 ± 0.4	1.5 ± 0.7	1.6 ± 0.5	1.7 ± 0.6	1.6 ± 0.5	1.5 ± 0.5

^*^Compared with Reponders group, *P* < 0.05.

**Table 3:** Rebound of different treatment regimen in stage two.

Symptoms	MF+PLA	MF+OXY	PLA+OXY	PLA+PLA
Nc FA	2.6 ± 0.4	2.0 ± 0.2*	2.1 ± 0.2*	2.7 ± 0.3
LA	1.9 ± 0.3*	0.7 ± 0.2*	1.9 ± 0.2*	2.5 ± 0.2
LS	2.6 ± 0.3#	1.3 ± 0.2*	2.6 ± 0.2#	2.6 ± 0.3
Sno FA	2.5 ± 0.3	1.9 ± 0.1*	1.9 ± 0.3*	2.6 ± 0.4
LA	1.7 ± 0.3*	0.8 ± 0.3*	1.8 ± 0.2*	2.7 ± 0.3
LS	2.7 ± 0.3#	1.2 ± 0.2*	2.6 ± 0.3#	2.5 ± 0.4
Rn FA	2.3 ± 0.3	2.4 ± 0.2	2.7 ± 0.2	2.7 ± 0.3
LA	1.3 ± 0.3*	0.8 ± 0.1*	2.5 ± 0.2	2.5 ± 0.3
LS	2.5 ± 0.2#	2.3 ± 0.2*^ ^#	2.6 ± 0.3	2.7 ± 0.2
Snz FA	2.4 ± 0.4	2.3 ± 0.2	2.5 ± 0.3	2.5 ± 0.3
LA	1.1 ± 0.3*	0.9 ± 0.2*	2.7 ± 0.2	2.5 ± 0.3
LS	2.5 ± 0.2#	2.2 ± 0.4*^ ^#	2.6 ± 0.4	2.6 ± 0.3
In FA	2.1 ± 0.3	2.2 ± 0.3	2.4 ± 0.2	2.3 ± 0.3
LA	1.2 ± 0.2*	0.8 ± 0.1*	2.3 ± 0.3	2.4 ± 0.2
LS	2.5 ± 0.4#	2.4 ± 0.2*^ ^#	2.3 ± 0.1	2.3 ± 0.3
Co FA	1.9 ± 0.4	2.0 ± 0.3	2.2 ± 0.3	2.1 ± 0.2
LA	1.3 ± 0.2*	0.9 ± 0.1*	2.1 ± 0.2	2.2 ± 0.3
LS	2.3 ± 0.3#	2.1 ± 0.3*^ ^#	2.1 ± 0.3	2.1 ± 0.3
TS FA	14.2 ± 1.5	12.2 ± 1.8*	13.6 ± 1.4*	15.5 ± 1.2
LA	8.8 ± 1.6*	4.8 ± 0.8*	13.8 ± 1.5*	14.9 ± 1.4
LS	15.3 ± 2.3#	12.9 ± 1.1*^ ^#	15.8 ± 1.6#	15.4 ± 1.3

Nc, nasal congestion; Sno, snoring; Rn, runny nose; Snz, sneezing; In, itchy nose; Co, cough; TS, total score; FA, first week active treatment; LA, last week treatment; LS, last study week.

^*^Compared with baseline score, *P* < 0.05.

^#^Compared with last week treatment, *P* < 0.05.

**Table 4:** Symptom scores after one week’s different treatment regimen in stage two.

Symptoms	MF + PLA	MF + OXY	PLA + OXY	PLA + PLA
Nasal congestion	2.6 ± 0.4	2.0 ± 0.2*	2.1 ± 0.2*	2.7 ± 0.3
Snoring	2.5 ± 0.3	1.9 ± 0.1*	1.9 ± 0.3*	2.6 ± 0.4
Runny nose	2.3 ± 0.3	2.2 ± 0.2	2.7 ± 0.2	2.7 ± 0.3
Sneezing	2.2 ± 0.4	2.1 ± 0.2	2.5 ± 0.3	2.5 ± 0.3
Itchy nose	2.1 ± 0.3	2.2 ± 0.3	2.4 ± 0.2	2.3 ± 0.3
Cough	1.9 ± 0.4	1.8 ± 0.3	2.0 ± 0.3	2.1 ± 0.2
Total score	14.2 ± 1.5	12.2 ± 1.8*	13.6 ± 1.4	15.5 ± 1.2

^*^Compared with baseline score, *P* < 0.05.
