# Supplementary material for: Combination of mometasone furoate and oxymetazoline for the treatment of adenoid hypertrophy concomitant with allergic rhinitis: A randomized controlled trial
Source: Sci Rep. 2017 Jan 18;7:40425. doi: 10.1038/srep40425 (PMC5241667; doi:10.1038/srep40425)
Supplement: Supplementary Figures [file srep40425-s1.pdf]

Combination of mometasone furoate and oxymetazoline for the treatment of adenoid hypertrophy concomitant with allergic rhinitis: A randomized controlled trial

Wenlong Liu*, PhD, Lifeng Zhou, MD, Qingxiang Zeng, PhD, Renzhong Luo,MD

From Department of Otolaryngology, Guangzhou Women and Children's Medical Center, Guangzhou Medical University, Guangzhou, China

Reprint requests: Wenlong Liu, PhD, Department of Otolaryngology, Guangzhou Women and Children's Medical Center, Guangzhou Medical University, No 9. Jinsui Road, Guangzhou, China,510623. Email:lwl20103@163.com, Phone:00862038076166, Fax: 00862038076166.

Figure legends

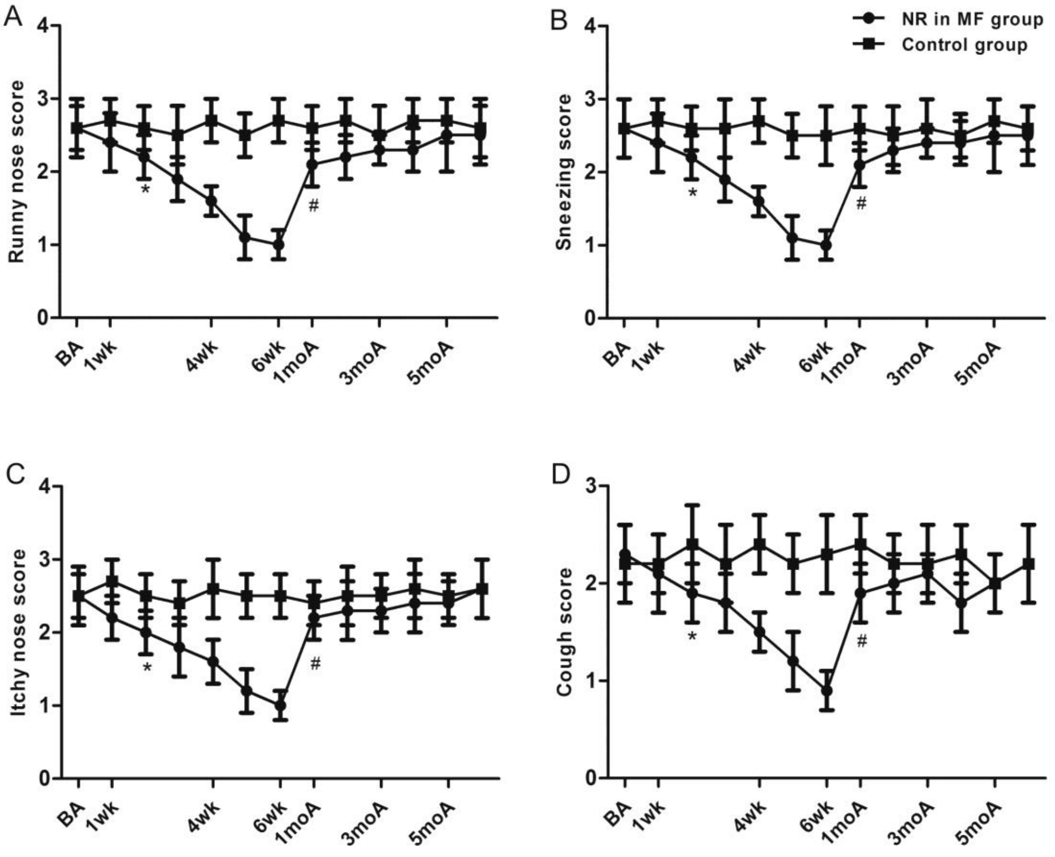

FIG. E1. Individual symptom scores of different time points between non-responders and control during the first treatment stage. A for runny nose score, B for sneezing score, C for itchy nose score and D for cough score. * Compared with baseline score, P<0.05. # P<0.05 versus the lowest score during treatment.

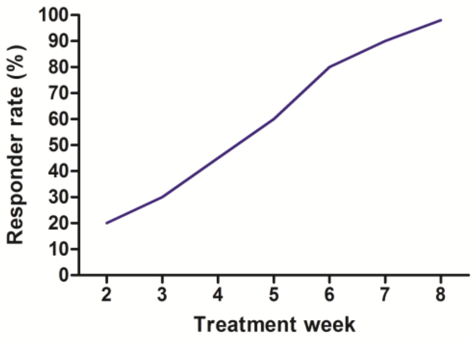

FIG. E2. Responder rate during different treatment weeks.

Table E1 Disposition of subjects and the reasons for discontinuation

	Stage 1		Stage 2			
Disposition summary	MF	Placebo	MF+Placebo	MF+OXY	Placebo+OXY	Placebo+Placebo
Screened subjects, no	120	120	17	17	17	17
Completed subjects, no	112	109	15	16	16	16
Discontinued subjects no	8	11	2	1	1	1
Reason for discontinuation						
Adverse event	1	1	0	0	1	0
Abnormal test result(s)	0	1	0	0	0	0
Treatment failure	0	0	0	0	0	0
Protocol violation	0	3	0	0	0	0
Noncompliance	2	2	1	0	0	0
Consent withdrawn	3	2	0	0	0	1
Lost to follow-up	2	2	1	1	0	0
Safety population, no. (%)	110 (99)	109 (100)	17 (100)	16 (95)	17 (100)	17 (100)
ITT population, no. (%)	111 (99)	109 (100)	17 (100)	16 (95)	17 (100)	17 (100)

ITT, Intent-to-treat.

*Safety population includes all randomized subjects who took at least 1 dose of the study drug.

#Intent-to-treat population includes all subjects who were randomized and had at least 1 postbaseline efficacy observation.

Table E2 Overview of treatment-related adverse events

	Stage 1		Stage 2			
	MF	Placebo	MF+Placebo	MF+OXY	Placebo+OXY	Placebo+Placebo
Number	110	109	15	16	16	16
Any adverse event	9	8	2	1	2	2
Dysgeusia	2	0	0	0	0	1
Epistaxis	2	2	1	0	0	0
Headache	1	**1**	0	1	1	1
Nasal discomfort	0	1	0	0	1	0
Nausea	1	2	1	0	0	0
Sneezing	2	1	0	0	0	0
Mucosal erosion	0	1	0	0	0	0
Somnolence	1	0	0	0	0	0
